# Development and Validation of a Clinical Score to Predict Neurological Outcomes in Patients With Out-of-Hospital Cardiac Arrest Treated With Extracorporeal Cardiopulmonary Resuscitation

**DOI:** 10.1001/jamanetworkopen.2020.22920

**Published:** 2020-11-24

**Authors:** Yohei Okada, Takeyuki Kiguchi, Taro Irisawa, Tomoki Yamada, Kazuhisa Yoshiya, Changhwi Park, Tetsuro Nishimura, Takuya Ishibe, Yoshiki Yagi, Masafumi Kishimoto, Toshiya Inoue, Yasuyuki Hayashi, Taku Sogabe, Takaya Morooka, Haruko Sakamoto, Keitaro Suzuki, Fumiko Nakamura, Tasuku Matsuyama, Norihiro Nishioka, Daisuke Kobayashi, Satoshi Matsui, Atsushi Hirayama, Satoshi Yoshimura, Shunsuke Kimata, Takeshi Shimazu, Shigeru Ohtsuru, Tetsuhisa Kitamura, Taku Iwami

**Affiliations:** 1 Department of Preventive Services, School of Public Health, Kyoto University, Kyoto, Japan; 2 Department of Primary Care and Emergency Medicine, Graduate School of Medicine, Kyoto University, Kyoto, Japan; 3 Critical Care and Trauma Center, Osaka General Medical Center, Osaka, Japan; 4 Department of Traumatology and Acute Critical Medicine, Osaka University Graduate School of Medicine, Suita, Japan; 5 Emergency and Critical Care Medical Center, Osaka Police Hospital, Osaka, Japan; 6 Department of Emergency and Critical Care Medicine, Kansai Medical University, Takii Hospital, Moriguchi, Japan; 7 Department of Emergency Medicine, Tane General Hospital, Osaka, Japan; 8 Department of Critical Care Medicine, Osaka City University, Osaka, Japan; 9 Department of Emergency and Critical Care Medicine, Kindai University School of Medicine, Osaka-Sayama, Japan; 10 Osaka Mishima Emergency Critical Care Center, Takatsuki, Japan; 11 Senshu Trauma and Critical Care Center, Osaka, Japan; 12 Senri Critical Care Medical Center, Saiseikai Senri Hospital, Suita, Japan; 13 Traumatology and Critical Care Medical Center, National Hospital Organization Osaka National Hospital, Osaka, Japan; 14 Emergency and Critical Care Medical Center, Osaka City General Hospital, Osaka, Japan; 15 Department of Pediatrics, Osaka Red Cross Hospital, Osaka, Japan; 16 Emergency and Critical Care Medical Center, Kishiwada Tokushukai Hospital, Osaka, Japan; 17 Department of Emergency and Critical Care Medicine, Kansai Medical University, Hirakata, Osaka, Japan; 18 Department of Emergency Medicine, Kyoto Prefectural University of Medicine, Kyoto, Japan; 19 Division of Environmental Medicine and Population Sciences, Department of Social and Environmental Medicine, Graduate School of Medicine, Osaka University, Osaka, Japan; 20 Public Health, Department of Social and Environmental Medicine, Osaka University Graduate School of Medicine, Osaka, Japan

## Abstract

**Importance:**

Extracorporeal cardiopulmonary resuscitation (ECPR) is expected to improve the neurological outcomes of patients with refractory cardiac arrest; however, it is invasive, expensive, and requires substantial human resources. The ability to predict neurological outcomes would assist in patient selection for ECPR.

**Objective:**

To develop and validate a prediction model for neurological outcomes of patients with out-of-hospital cardiac arrest with shockable rhythm treated with ECPR.

**Design, Setting, and Participants:**

This prognostic study analyzed data from the Japanese Association for Acute Medicine Out-of-Hospital Cardiac Arrest registry, a multi-institutional nationwide cohort study that included 87 emergency departments in Japan. All adult patients with out-of-hospital cardiac arrest and shockable rhythm who were treated with ECPR between June 2014 and December 2017 were included. Patients were randomly assigned to the development and validation cohorts based on the institutions. The analysis was conducted between November 2019 and August 2020.

**Exposures:**

Age (<65 years), time from call to hospital arrival (≤25 minutes), initial cardiac rhythm on hospital arrival (shockable), and initial pH value (≥7.0).

**Main Outcomes and Measures:**

The primary outcome was 1-month survival with favorable neurological outcome, defined by Cerebral Performance Category 1 or 2. In the development cohort, a simple scoring system was developed to predict this outcome using a logistic regression model. The diagnostic ability and calibration of the scoring system were assessed in the validation cohort.

**Results:**

A total of 916 patients were included, 458 in the development cohort (median [interquartile range {IQR}] age, 61 [47-69] years, 377 [82.3%] men) and 458 in the validation cohort (median [IQR] age, 60 [49-68] years; 393 [85.8%] men). The cohorts had the same proportion of favorable neurological outcome (57 patients [12.4%]). The prediction scoring system was developed, attributing a score of 1 for each clinical predictor. Patients were divided into 4 groups, corresponding to their scores on the prediction model, as follows: very low probability (score 0), low probability (score 1), middle probability (score 2), and high probability (score 3-4) of good neurological outcome. The mean predicted probabilities in the groups stratified by score were as follows: very low, 1.6% (95% CI, 1.6%-1.6%); low, 4.4% (95% CI, 4.2%-4.6%); middle, 12.5% (95% CI, 12.1%-12.8%); and high, 30.8% (95% CI, 29.1%-32.5%). In the validation cohort, the *C* statistic of the scoring system was 0.724 (95% CI, 0.652-0.786). The predicted probability was evaluated as well calibrated to the observed favorable outcome in both cohorts by visual assessment of the calibration plot.

**Conclusions and Relevance:**

In this study, the scoring system had good discrimination and calibration performance to predict favorable neurological outcomes of patients with out-of-hospital cardiac arrest and shockable rhythm who were treated with ECPR.

## Key Points

**Question:**

Can the neurological outcome of patients with out-of-hospital cardiac arrest and shockable rhythm who are treated with extracorporeal cardiopulmonary resuscitation (ECPR) be predicted using accessible information?

**Findings:**

In this prognostic study of 916 patients, a model using time to hospital arrival, pH in initial blood gas assessment, shockable rhythm on hospital arrival, and being younger than 65 years was developed to predict survival with good neurological outcome. The model had good performance and was well calibrated.

**Meaning:**

These findings suggest that this model may be useful for predicting the neurological outcomes of patients with out-of-hospital cardiac arrest and shockable rhythm treated with ECPR.

## Teaser

This prognostic study develops and validates a prediction model for neurological outcomes of patients with out-of-hospital cardiac arrest with shockable rhythm treated with extracorporeal cardiopulmonary resuscitation.

## Introduction

Extracorporeal cardiopulmonary resuscitation (ECPR) is a potentially favorable option for patients with out-of-hospital cardiac arrest (OHCA) and refractory ventricular fibrillation (VF).^1,2,3,4,5^ However, this method is invasive, expensive, and requires substantial human resources.^1,2,3,6,7^ Therefore, it is essential to assess appropriate candidates immediately after hospital arrival.

In a previous systematic review,^8^ several factors, such as initial shockable rhythm during resuscitation (odds ratio [OR], 2.29; 95% CI, 1.53-3.42), have been reported to be associated with the outcome after OHCA; however, relative effect measurements, such as odds ratios, cannot be directly interpreted and used to decide a treatment strategy in the clinical setting. Rather, the actual probability or the predictive accuracy (eg, the likelihood ratio) for the outcome is necessary to support decision-making. Therefore, clinical prediction rules, such as the CHADS_2_ (congestive heart failure, hypertension, age ≥75 years, diabetes, and stroke [doubled])^9^ score for atrial fibrillation and the CURB-65 (confusion, urea, respiratory rate, blood pressure, and age ≥65 years)^10^ for pneumonia, have been used to simply and quickly estimate the probability of outcome. Similarly, a clinical prediction rule is also necessary to estimate the neurological outcomes in patients with OHCA treated with ECPR. The present study aimed to develop and validate a prediction rule for the neurological outcomes among patients with OCHA and shockable rhythm who are treated with ECPR.

## Methods

The methods of this study have been reported according to the Transparent Reporting of a Multivariable Prediction Model for Individual Prognosis or Diagnosis (TRIPOD) reporting guideline.^11^ The ethics committee of Kyoto University approved this study with a waiver of informed consent because of the retrospective nature of the analysis with minimal risk to patients.

### Data Source

We performed a retrospective analysis of the Japanese Association for Acute Medicine Out-of-Hospital Cardiac Arrest (JAAM-OHCA) registry.^12^ This registry is a prospective, multicenter, nationwide database established by the committee of the JAAM-OHCA Registry, and the details of the registry have been described previously.^12,13^ Briefly, the registry includes prehospital information, in-hospital information, and outcome among patients with OHCA transported to emergency departments of 87 institutions (66 university hospitals and/or tertiary critical care centers) in Japan. The tertiary critical care centers are certified by the Ministry of Health, Labor, and Welfare in Japan, and they are required to provide highly specialized treatment, such as ECPR, percutaneous coronary intervention, and targeted temperature management for 24 hours a day. The other 21 hospitals are not certified as critical care centers, but they provide emergency medical services to the community, and some can provide ECPR and intensive care. In total, 34 754 patients with OHCA were registered between June 2014 and December 2017 in the JAAM-OHCA registry.

Prehospital information was collected by paramedics based on the standardized Utstein format,^14^ and it was verified by the Fire and Disaster Management Agency of Japan. In-hospital information, such as treatment in the emergency department and after admission to the intensive care unit (ICU), was registered by the clinicians or clinical data administrators in each institution using electrical data capturing with the standardized reporting form.^12,13^ The JAAM-OHCA registry committee checked the quality of the in-hospital and prehospital data logically. Finally, the deidentified data were provided to the researchers by the committee.

### Participants

We included all adult (aged ≥18 years) patients with OHCA and shockable rhythm from the time of collapse to hospital arrival who had been treated with ECPR between June 2014 and December 2017. The shockable rhythm was defined as VF or pulseless ventricular tachycardia. The shockable rhythm from the time of collapse to hospital arrival was defined as defibrillated by the bystander on the site or confirmed by the paramedics or clinicians on hospital arrival. ECPR was defined as an emergency cannulation of veno-arterial extracorporeal membrane oxygenation for patients with OHCA who sustained cardiac arrest at the time of arrival at the hospital.

The decision to perform ECPR was determined by the individual physician caring for each patient. We excluded patients who received no resuscitation in the hospital, such as those with rigor mortis or those who had obtained do not attempt resuscitation orders. In addition, the following patients were also excluded from the study: patients with OHCA who were transported to the participating intuitions after receiving any treatments in other hospitals; patients with traumatic cardiac arrest; patients with no prehospital data; patients who had return of spontaneous resuscitation (ROSC) on hospital arrival; and patients who opted out of the study. ROSC was defined as the presence of palpable pulse for more than 30 seconds.^15^

### Development and Validation Cohorts

The included institutions were randomly divided into 2 cohorts based on outcome. One cohort was used to develop the prediction model (the development cohort), and the other was used to validate and assess the diagnostic abilities of the model (the validation cohort).^16,17^

### Outcome

The primary outcome of our study was 1-month survival with favorable neurological outcome, defined by Cerebral Performance Category (CPC) 1 or 2.^14^ Details appear in eAppendix 1 in the Supplement.

### Predictors

Based on the previous studies^8,18,19,20,21,22,23,24,25,26^ and the opinion of the emergency medicine experts in our research group (Y.O., T. Kiguchi, T.I., T.Y., T.M., T.N., M.K., N.N., S.Y., S.K., S.O., T. Kitamura, and T.I.), we selected the following variables as potential predictors of in-hospital mortality: age, witnessed by bystander, CPR by bystander, shockable rhythm on hospital arrival, time from emergency call to hospital arrival, and initial serum pH value. To ensure that the model is user friendly, especially for emergency settings, we categorized the potential predictors based on their round value or commonly used ranges.

### Sample Size Estimation

There is no generally accepted approach for the estimation of the sample size for derivation of risk prediction models. Although we understand that it is controversial, it has been suggested to have at least 10 events per candidate variable for the derivation of a model, and this approach has been widely adopted.^27^ We selected the predictors based on this rule.

### Data Measurement, Collection, and Handling Missing Data

We obtained the clinical information from the database, and it was categorized as follows: sex, age (18-65 years, 65-74 years, ≥75 years), witnessed by bystander, CPR performed by bystander, prehospital initial cardiac rhythm (shockable or nonshockable and other), cardiac rhythm on hospital arrival (shockable or nonshockable), pH in the blood gas assessment initially measured after hospital arrival, resuscitation time course, and outcome. The age category was defined based on the government reference.^28^ The resuscitation time course was defined as the time from the emergency call for an ambulance to hospital arrival, blood collection for gas assessment, and the start of ECPR in the hospital. Missing values were shown as missing or unknown, and a complete case analysis was conducted.

### Statistical Analysis

#### Patient and Hospital Characteristics

The patient characteristics have been already described. Furthermore, we added the hospital’s basic information, including type (tertiary care center or not) and number of beds. Tertiary center was defined as a university hospital and/or tertiary critical care center certified by the government, as explained earlier. Data are shown as medians and interquartile ranges (IQRs) for continuous variables, and as numbers and percentages for categorical variables.

#### Development and Evaluation of the Prediction Model

In the development cohort, we calculated the crude ORs with 95% CIs of the predictor candidates for the outcome using univariate logistic analysis. We selected predictors to develop a parsimonious model based on the results of the univariate analysis (*P* < .05) and expert opinion.^27^ Subsequently, we applied a multivariable logistic regression model to generate the β coefficient with SE for each variable. The performance of the model was evaluated based on Somers Dxy rank correlation, *C* index, Nagelkerke *R*^2^ value, calibration intercept and slope, and Brier score.^27^ Further, we performed the Hosmer-Lemeshow goodness-of-fit test to assess the calibration in the development cohort.^16^ Calibration plots were created to graphically indicate the association between the predicted and observed outcome using locally weighted scatterplot smoothing.^16^ We used a bootstrapping procedure (200 samples drawn with replacement from the original sample) to assess the internal validation of the model.^16^

#### Score and Grouping

Subsequently, we developed a scoring system to predict the outcome using a simple integer based on each variable’s β coefficient in the development cohort.^16^ The diagnostic abilities (ie, sensitivity, specificity, positive likelihood ratio, and negative likelihood ratio) of each score were calculated. Appropriate cutoff values were set for a rule-in and rule-out approach to help in decision-making, and the patients were divided into 4 groups.

#### Validation and Evaluation of the Prediction Model, Scoring, and Grouping

For external validation, the developed model was applied to the validation cohort, and the discrimination and calibration performances were described. The relationships between predicted and observed outcome in each group were indicated for calibration. Furthermore, we performed decision curve analysis to indicate the net benefit and clinical utility of the score.^29^ The net benefit and decision curve analysis is detailed in eAppendix 2 in the Supplement. Results with a 2-sided *P* < .05 were considered statistically significant. Statistical analyses were performed between November 2019 and August 2020 using the JMP Pro version 14 software (SAS Institute Inc) and R version 1.1.456 (R Project for Statistical Computing) with the rms and rmda packages.^30^

## Results

### Study Participants

**Figure 1.**
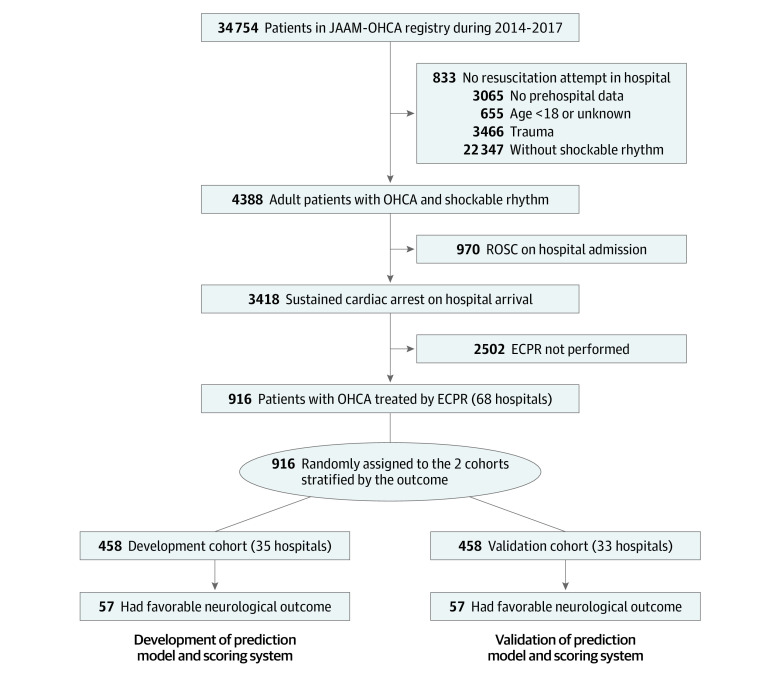
Study Flowchart ECPR indicates extracorporeal cardiopulmonary resuscitation; JAAM-OHCA, Japanese Association for Acute Medicine Out-of-Hospital Cardiac Arrest; and ROSC, return of spontaneous resuscitation.

Among 34 754 patients in the JAAM-OHCA database, we included 916 who met the eligibility criteria; 458 in the development cohort (from 35 hospitals; median [IQR] age, 61 [47-69] years; 377 [82.3%] men) and 458 in the validation cohort (from 33 hospitals; median [IQR] age, 60 [49-68] years; 393 [85.8%] men) (Figure 1). Additional patient characteristics and in-hospital data are described in Table 1 and eTable 1 in the Supplement. Most patients had their collapse witnessed by a bystander (development: 344 [75.1%]; validation: 367 [80.1%]) and received defibrillation from paramedics (development: 429 [94.7%]; validation: 434 [95.6%]). Nearly half of patients received CPR from bystanders (development: 223 [48.7%]; validation: 226 [49.3%]). The favorable neurological outcome in the development and validation cohorts were 12.4% (57) and 12.4% (57), respectively.

**Table 1.** Patient Characteristics

Characteristic	Patients, No. (%), by cohort			
	Development (n = 458)		Validation (n = 458)	
	Favorable outcome (n = 57)	Unfavorable outcome (n = 401)	Favorable outcome (n = 57)	Unfavorable outcome (n = 401)
Patient information				
Men	46 (10.0)	331 (72.3)	44 (9.6)	349 (76.2)
Age, y				
Median (IQR)	51 (42-65)	62 (48-70)	54 (49-67)	61 (49-69)
18-64	43 (75.4)	230 (57.4)	40 (70.2)	247 (61.6)
65-74	12 (21.1)	105 (26.2)	14 (24.6)	112 (27.9)
≥75	2 (3.5)	66 (16.5)	3 (5.3)	42 (10.5)
Witnessed	46 (80.7)	298 (74.3)	51 (89.5)	316 (78.8)
Bystander CPR	31 (54.4)	192 (47.9)	28 (49.1)	198 (49.4)
Shock by bystander	10 (17.5)	27 (6.7)	6 (10.5)	39 (9.7)
Shock by paramedics	53 (93.0)	376 (93.8)	56 (98.3)	378 (95.2)
Initial rhythm at the scene				
Shockable	45 (79.0)	313 (78.1)	51 (89.5)	320 (79.8)
Nonshockable or other	12 (21.1)	88 (22.0)	6 (10.5)	81 (20.2)
Initial rhythm on hospital arrival				
Shockable	47 (82.5)	225 (56.1)	50 (87.7)	225 (56.1)
Nonshockable	10 (17.5)	176 (43.9)	7 (12.3)	176 (43.9)
Time from call to hospital arrival, min				
Median (IQR)	30 (23-39)	32 (26-39)	28 (22-33.75)	33 (26-41)
≤25	21 (36.8)	77 (19.2)	25 (43.9)	94 (23.4)
26-35	16 (28.1)	188 (46.9)	19 (33.3)	145 (36.2)
35-45	9 (15.8)	86 (21.5)	6 (10.5)	81 (20.2)
>45	8 (14.0)	48 (12.0)	6 (10.5)	69 (17.2)
Missing	3 (5.3)	2 (0.5)	1 (1.8)	12 (3.0)
Treated by tertiary center	56 (98.3)	373 (93.0)	56 (98.3)	400 (99.8)
Initial pH on hospital arrival				
Median (IQR)	7.01 (6.88-7.12)	6.93 (6.82-7.03)	7.01 (6.81-7.1)	6.92 (6.83-7.04)
≥7.0	26 (45.6)	118 (29.4)	30 (52.6)	122 (30.4)
6.9-7.0	12 (21.1)	102 (25.4)	7 (12.3)	102 (25.4)
6.8-6.9	8 (14.0)	88 (21.9)	7 (12.3)	92 (22.9)
<6.8	6 (10.5)	73 (18.2)	13 (22.8)	68 (17.0)
Missing	5 (8.8)	20 (5.0)	0	17 (4.2)
Time, median (IQR), min				
From call to the blood gas	48 (36-63)	43 (35-56)	40 (30-62)	44 (34-57)
From call to ECPR start	51 (43-74)	58 (48-71)	52 (43-62)	58 (49-73)

Abbreviations: CPR, cardiopulmonary resuscitation; ECPR, extracorporeal cardiopulmonary resuscitation; IQR, interquartile range.

### Model Development and Performance

In the development cohort, we calculated the crude OR with 95% CIs of the predictor candidates (eTable 2 in the Supplement) and selected the predictors for the model as follows: younger than 65 years; pH, at least 7.0; time from emergency call to hospital arrival, 25 minutes or less; and shockable rhythm on hospital arrival. We calculated the β coefficient and SE using the logistic regression analysis (Table 2). The *C* statistic of the model was 0.753 (95% CI, 0.687-0.809). The bias-corrected *C* statistic by bootstrapping was 0.738 (95% CI, 0.672-0.802). The equation of the prediction model to calculate the probability of the outcome, other performances, and the calibration plot are described eTable 3 and eFigure 1 in the Supplement. The calibration plot and results of Hosmer-Lemeshow test (χ^2^_8_ = 5.57; *P* = .70) showed that the prediction was well calibrated to the observation; however, bootstrapping indicated slight overestimation in the high-probability group.

**Table 2.** Multivariable Logistic Model for the Neurological Outcome

Variable	β coefficient (SE)	*P* value
Intercept	−4.14 (0.48)	<.001
Time from call to hospital arrival, ≤25 min	1.04 (0.31)	<.001
pH, ≥7.0	0.75 (0.31)	.01
Shockable rhythm on hospital arrival	1.35 (0.37)	<.001
Age, 18-64 y	0.95 (0.34)	.005

We created a simple scoring system based on the β coefficient (Table 3). The scoring system was summarized by the mnemonic TiPS65 (Table 3): Ti, time from call to hospital arrival, 25 minutes or less; P, pH value, ≥7.0; S, shockable rhythm on hospital arrival; and 65, age 65 years or younger. The *C* statistic of the score was 0.741 (95% CI, 0.682-0.792). The diagnostic abilities (sensitivity, specificity, negative likelihood ratio, and positive likelihood ratio) are described in eTable 3 and eTable 4 in the Supplement. Based on the scoring, we divided the patients into 4 groups, as follows: very low probability (score 0), low probability (score 1), middle probability (score 2), and high probability (score 3-4) of good neurological outcome.

**Table 3.** TiPS65 Scoring System

Variable	Score
Time from call to hospital arrival ≤25 min	1
pH ≥7.0	1
Shockable on hospital arrival	1
<65 y	1
Sum	4

Abbreviation: TiPS65, time to hospital arrival, pH in initial blood gas assessment, shockable rhythm on hospital arrival, and age <65 years.

### External Validation of the Model

**Figure 2.**
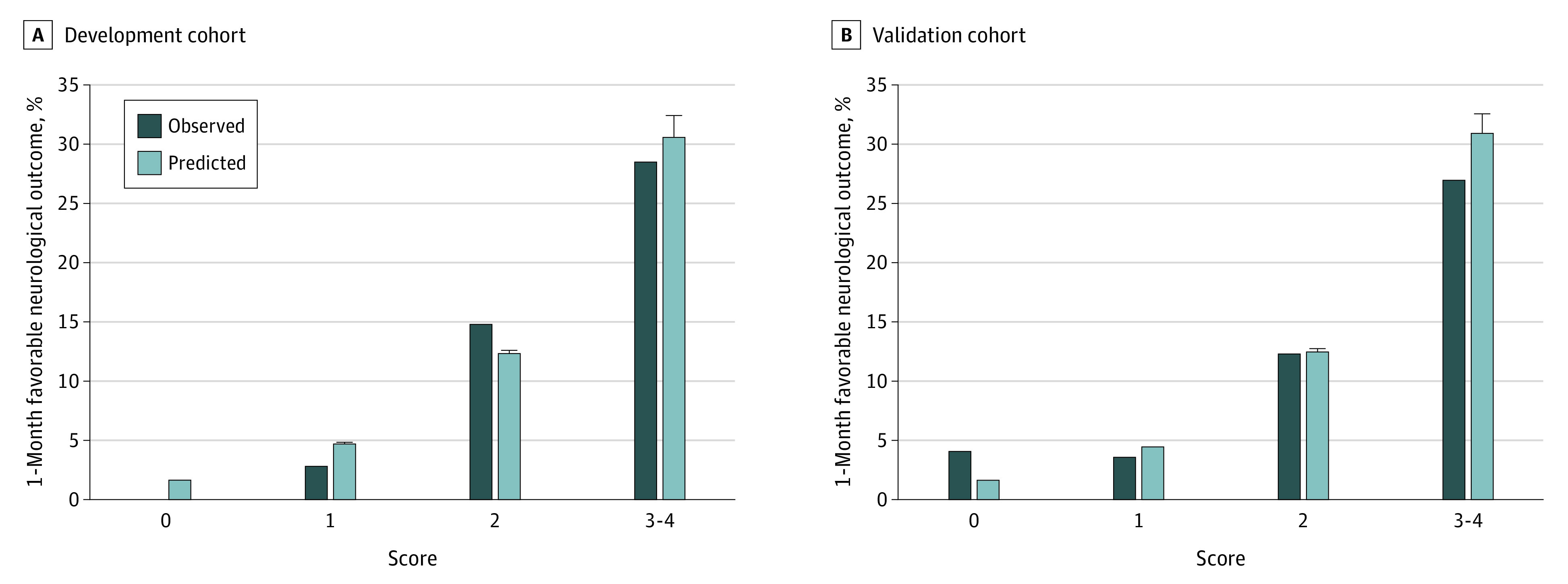
Predicted Probability and Observed 1-Month Favorable Neurological Outcome in the Development and Validation Cohorts Observed category indicates actual number of favorable outcomes divided by the total patients in each group, while the predicted category is the mean predicted probability, with 95% CIs represented by error bars. Predicted probabilities were calculated using the logistic model described in Table 2.

In the validation cohort, the *C* statistic of the prediction model was 0.731 (95% CI, 0.653-0.797) and that of the scoring system was 0.724 (95% CI, 0.652-0.786). The diagnostic abilities (sensitivity, specificity, negative likelihood ratio, and positive likelihood ratio) are described in eTable 5 in the Supplement. The mean predicted probabilities in the groups stratified by TiPS65 score were as follows: very-low (score 0), 1.6% (95% CI, 1.6%-1.6%); low (score 1), 4.4% (95% CI, 4.2%-4.6%); middle (score 2), 12.5% (95% CI, 12.1%-12.8%); and high (score 3-4), 30.8% (95% CI, 29.1%-32.5%) (Figure 2). The mean predicted probabilities with 95% CIs and the observed outcome in the development and validation cohorts are summarized in Figure 2. The predicted probability was well calibrated to the observed favorable outcome in both cohorts. Furthermore, in the validation cohort, we calculated the net benefit and indicated the decision curve analysis (eFigure 2 in the Supplement), which demonstrated that the net benefit of using the score was larger than those of all ECPR strategies or no ECPR strategy. Therefore, our model may be helpful for selecting appropriate candidates for ECPR.

## Discussion

In the present study, using the nationwide OHCA database in Japan, the TiPS65 scoring system was helpful in predicting favorable neurological outcome after shockable OHCA in patients who were treated with ECPR. Based on the scoring system, it would appear to be reasonable to give ECPR to patients with OHCA in the high-probability group (score 3-4; predicted favorable outcome, 30.8%; 95% CI, 29.1%-32.5%), and to reconsider ECPR in the very-low probability group (score 0; predicted favorable outcome, 1.6%; 95% CI, 1.6%-1.6%).

This study has several strengths and clinical implications. First, the score may be helpful for clinicians to easily predict the probability of good neurological outcome and to assist them in decision-making for ECPR. If the score is greater than 3, the probability of good neurological outcome is approximately 30% among patients with OHCA and shockable rhythm who are treated with ECPR. We believe that this probability is high enough to attempt ECPR. On the contrary, if the score is 0, the probability is approximately 1%. Owing to its invasiveness and cost, it would be reasonable to consider the indication of ECPR carefully in these patients.^7^ Second, the decision curve analysis indicated that the net benefit of using the score was higher than when ECPR was performed in all patients. This means that the score can potentially help to avoid the unfavorable implementation of ECPR. Thus, it may also contribute to the appropriate use of medical resources in health policy. Third, this scoring system can be used as soon as patients present to the emergency department because age, time, and initial rhythm are generally available immediately after the patient arrives at the hospital. Furthermore, the pH value is soon available after the hospital arrival. Fourth, this model has been validated by bootstrapping and the validation cohort. Thus, it may be applicable to similar settings. Lastly, this information may be useful when considering eligible patients for studies assessing the efficacy of ECPR. For example, patients with 0 points on the TiPS65 score have a very low probability of 1-month favorable neurological outcomes, indicating that ECPR may be ineffective in this population. Thus, it would be reasonable to exclude them when planning a randomized clinical trial to assess the efficacy of the method. Considering these strengths, this score would be helpful for patient selection in the actual clinical setting, cost-effective medical resource allocation, and assessment of ECPR efficacy in future studies.

Age is known to be among the most critical factors associated with neurological outcomes among patients with OHCA.^31,32,33^ The sustained shockable rhythm at the time of hospital arrival has also been reported to be associated with neurological outcome.^24^ In patients with sustained shockable rhythm, oxygen delivery and myocardial energy substrates may persist^34^; as a result, adequate perfusion of the injured myocardium by ECPR may lead to a higher chance of ROSC and organ recovery. Moreover, the pH value is representative of respiratory and metabolic acidosis, which are influenced by the duration of low-flow time and the quality of chest compression.^8,19,20,21,22,23^ Prehospital time is also expected to be associated with the length of low-flow time.^18,25^ Therefore, the scoring system proposed in this study can be explained by these factors.

### Limitations

This study has several limitations. First, the study has the risk of selection bias. The cohort in our study comprised OHCA patients with shockable rhythm who were treated by ECPR. However, ECPR was selected not based on the unified criteria but by each clinician’s judgment. Although we believe that most patients in our study were reasonable candidates for ECPR based on their characteristics, the imperfect inclusion criteria lead to concerns about generalizability. Second, even though the registered data were double-checked by clinicians or data administrators and the JAAM-OHCA committee members, inaccurate measurement, insufficient records, and missing data occasionally happen in acute care settings, such as during resuscitation. This may have led to measurement bias. Third, with respect to outcomes, although this study included a relatively large sample size, the number of outcomes was limited. Consequently, the model may increase the risk of overfitting, which is a modeling error to fit the statistical model with too many degrees of freedom in the modeling process. Furthermore, although this model was considered well calibrated by the calibration plot, the overall performance and discrimination ability were considered fair based on the *C* statistics and Brier score, which may reduce the applicability of the model.^16^ Fourth, the JAAM-OHCA database does not include complete clinical details, such as clinical course after the admission, or details of ECPR procedures. Fifth, the utility of this prediction model in an actual clinical setting is unclear. Therefore, we suggest that clinicians judge the indication of ECPR not only using this score but also on the basis of other clinical factors. Sixth, although ECPR is expected to be beneficial as a last resort to save patients with refractory VF, the method has not been assessed by randomized clinical trials, and it remains controversial.^2,4,5,35,36,37^ It must be noted that the utility of the score is based on the assumption that ECPR is effective among patients with refractory OHCA. Further prospective research is needed to evaluate the utility and validity of our scoring system in other clinical settings.

## Conclusions

This study found that the TiPS65 scoring system may be helpful in predicting favorable neurological outcomes among patients with OHCA and shockable rhythm treated with ECPR. Further studies are needed to prospectively assess our scoring system with additional refinements, validate it in different settings, and elucidate its clinical usefulness.

## Notes

Supplement. **eAppendix 1. **Definition of the Outcome **eAppendix 2. **Net Benefit and Decision Curve Analysis **eTable 1. **Patient Characteristics by Cohorts **eTable 2. **Crude Odds Ratio of Predictor Candidates **eTable 3. **Model Performance and Internal Validation **eFigure 1. **Calibration Plot of the Model in Development Cohort **eTable 4. **Diagnostic Ability of the Score in Validation Cohort **eTable 5.** Diagnostic Ability of the Score Group in Validation Cohort **eFigure 2. **Decision Curve Analysis **eReferences.**
