# Supplementary material for: Development and Validation of a Clinical Score to Predict Neurological Outcomes in Patients With Out-of-Hospital Cardiac Arrest Treated With Extracorporeal Cardiopulmonary Resuscitation
Source: JAMA Netw Open. 2020 Nov 24;3(11):e2022920. doi: 10.1001/jamanetworkopen.2020.22920 (PMC7686862; doi:10.1001/jamanetworkopen.2020.22920)
Supplement: Supplement. — eAppendix 1. Definition of the Outcome eAppendix 2. Net Benefit and Decision Curve Analysis eTable 1. Patient Characteristics by Cohorts eTable 2. Crude Odds Ratio of Predictor Candidates eTable 3. Model Performance and Internal Validation eFigure 1. Calibration Plot of the Model in Development Cohort eTable 4. Diagnostic Ability of the Score in Validation Cohort eTable 5. Diagnostic Ability of the Score Group in Validation Cohort eFigure 2. Decision Curve Analysis eReferences. [file jamanetwopen-e2022920-s001.pdf]

Supplemental Online Content

Okada Y, Kiguchi T, Irisawa T, et al. Development and validation of a clinical score to predict neurological outcomes in patients with out-of-hospital cardiac arrest treated with extracorporeal cardiopulmonary resuscitation. *JAMA Netw Open*. 2020;3(11):e2022920. doi:10.1001/jamanetworkopen.2020.22920

eAppendix 1. Definition of the Outcome
eAppendix 2. Net Benefit and Decision Curve Analysis
eTable 1. Patient Characteristics by Cohorts
eTable 2. Crude Odds Ratio of Predictor Candidates
eTable 3. Model Performance and Internal Validation
eFigure 1. Calibration Plot of the Model in Development Cohort
eTable 4. Diagnostic Ability of the Score in Validation Cohort
eTable 5. Diagnostic Ability of the Score Group in Validation Cohort
eFigure 2. Decision Curve Analysis
eReferences.

This supplemental material has been provided by the authors to give readers additional information about their work.

© 2020 Okada Y et al. *JAMA Network Open.*

eAppendix 1. Definition of the Outcome

The primary outcome of our study was one-month survival with favorable neurological outcome defined by Cerebral Performance Category (CPC) 1 or 2. CPC is commonly used to evaluate the neurological status after OHCA according to the following criteria: category 1, good cerebral performance; category 2, moderate cerebral disability; category 3, severe cerebral disability; category 4, coma or vegetative state; and category 5, death/brain death.[1]

The Fire and Disaster Management Agency of Japan requires emergency medical service personnel to ask the clinicians follow-up and confirm the patients' outcomes using the Utstein-style reporting template. The CPC was evaluated by the clinicians in each hospital and was registered in the database by the fire departments. Although the details of the follow-up were not strictly determined, in general, if the patient stayed in the hospital, the outcome was evaluated by the clinician responsible for the patient. If the patient was discharged, the outcome was assessed in the out-patient department or based on referral documents from the transferred hospitals.

© 2020 Okada Y et al. *JAMA Network Open.*

eAppendix 2. Net Benefit and Decision Curve Analysis

Net-benefit refers to the difference between benefit and weighted harm of the test calculated as:

$$\text{Net-benefit} = \text{proportion of TP} - \text{proportion of FP} \times \text{weighting}$$

$$\text{Weighting} = p / (1 - p)$$

where, p is threshold probability. Net-benefit refers to the number of FP patients that have clinical importance equal to one TP patient; threshold probability refers to the level of diagnostic certainty above which the patient would be treated based on hospital policy or own preference. For example, if "$p = 0.1$," the weighting = $0.1 / (1 - 0.1) = 1/9$, meaning that 9 FP is equal to 1 TP, so if 10% of patients are TP (9 FP and 1 TP), all patients should be treated. In general, in decision curve analysis, net-benefit is plotted using index test or prediction under several thresholds of probability. Further, net-benefit is plotted if all patients are treated as positive or negative regardless of the index test result or prediction. Decision curve analysis can help obtain the highest net-benefit. Further reference is available in some previous literatures.[2-4]

In this study, we either assumed all patients to be positive and performed ECPR (All ECPR strategy) as actual situation or considered all patients to be negative and performed no ECPR and unfavorable outcome regardless of the score.

© 2020 Okada Y et al. *JAMA Network Open.*

eTable 1. Patient Characteristics by Cohorts

Variables		Development cohort (N=458)		Validation cohort (N=458)	
**Patients Information**					
**Sex**	Men	377	82.3%	393	85.8%
**Age**		61 [47-69]		60 [49-68]	
	18-64	273	59.6%	287	62.7%
	65-74	117	25.5%	126	27.5%
	≥75	68	14.8%	45	9.8%
**Witnessed**		344	75.1%	367	80.1%
**Bystander CPR**		223	48.7%	226	49.3%
**Shock performed by bystander**		37	8.1%	45	9.8%
**Shock performed by paramedics**		429	94.7%	434	95.6%
**Initial rhythm at the scene**					
	Shockable	358	78.2%	371	81.0%
	Non-shockable/ Other	100	21.8%	87	19.0%
**Initial rhythm on hospital arrival**					
	Shockable	272	59.4%	275	60.0%
	Non-Shockable	186	40.6%	183	40.0%
**Time from E-call to hospital arrival**		32 [26-39]		32 [25-41]	
	≤25min	98	21.4%	119	26.0%
	26-35	204	44.5%	164	35.8%
	35-45	95	20.7%	87	19.0%
	>45	56	12.2%	75	16.4%
	missing	5	1.1%	13	2.8%
**Treated by Tertiary center**		429	93.7%	456	99.6%
**Initial pH on hospital arrival**		6.93 [6.83-7.03]		6.93 [6.83-7.05]	
	≥7	144	31.4%	152	33.2%
	6.9-7	114	24.9%	109	23.8%
	6.8-6.9	96	21.0%	99	21.6%
	<6.8	79	17.2%	81	17.7%
	Missing	25	5.5%	17	3.7%
**Time from E-call to the blood gas**		44 [35-56]		43.5 [33-58]	
**Time from E-call to ECPR start**		57 [48-71]		58 [48-71]	

Continuous variables are described as median [interquartile range]. Categorical variables are described as number (%). CPR: cardiopulmonary resuscitation, Initial rhythm at the scene: Initial rhythm confirmed by paramedics at the scene, Call: emergency call for ambulance, ECPR: extracorporeal cardiopulmonary resuscitation

© 2020 Okada Y et al. *JAMA Network Open.*

eTable 2. Crude Odds Ratio of Predictor Candidates

	Crude OR	95%CI		p-value
Age				
18-64 /75≤	6.17	1.46	26.14	0.014
65-74 /75≤	3.77	0.82	17.39	0.089
Witnessed (Yes/No)	1.45	0.72	2.90	0.299
Bystander CPR performed (Yes / No)	1.30	0.74	2.26	0.359
Initial rhythm shockable (Yes / No)	1.05	0.53	2.08	0.879
Shockable on hospital arrival (Yes / No)	3.68	1.81	7.48	<0.001
Time from call to hospital arrival				
≤25 min/ >25	2.96	1.66	5.30	<0.001
≤30 min/ >30	1.39	0.80	2.43	0.244
≤35 min/ >35	1.05	0.59	1.88	0.861
Initial pH on hospital arrival				
≥7.0 / <7.0	2.01	1.14	3.53	0.015
≥6.9 / <6.9	1.65	0.92	2.95	0.095
≥6.8 / <6.8	1.26	0.62	2.54	0.512

OR: odds ratio, CI: confidence interval, CPR: cardiopulmonary resuscitation, Call: emergency call for ambulance, "Missing" is included in reference.

© 2020 Okada Y et al. *JAMA Network Open.*

eTable 3. Model Performance and Internal Validation

Parameter	Original	Bias-corrected	n
Dxy	0.506	0.479	200
C-statistics	0.753	0.739	200
Nagelkerke R^2	0.163	0.142	200
Intercept	0	-0.109	200
Slope	1	0.931	200
Brier score	0.100	0.102	200

Intercept: Calibration intercept, Slope: Calibration slope [5]

Model performance and internal validation were assessed by bootstrap (n=200) in development cohort.

© 2020 Okada Y et al. *JAMA Network Open.*

eFigure 1. Calibration Plot of the Model in Development Cohort

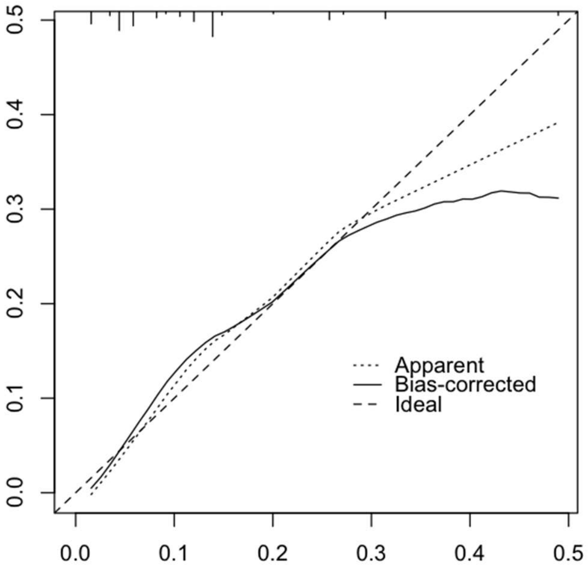

X-axis: Predicted probability, Y-axis: Observed, Apparent: original model, Bias-corrected: bias corrected by bootstrapping. Original prediction model is evaluated by bootstrapping (n=200) in development cohort.

This model is described in Table 2 of the main text.

© 2020 Okada Y et al. *JAMA Network Open.*

eTable 4. Diagnostic Ability of the Score in Validation Cohort

Score	Sp	Se	LR+	LR-	TP	TN	FP	FN
4	0.985	0.193	12.90	0.82	11	395	6	46
3	0.810	0.491	2.59	0.63	28	325	76	29
2	0.399	0.895	1.49	0.26	51	160	241	6
1	0.060	0.982	1.04	0.29	56	24	377	1
0	-	-	-	-	57	0	401	0

Sp: Specificity, Se: Sensitivity, LR+: Positive likelihood ratio, LR-: Negative likelihood ratio, TP: true-positive, TN: true-negative, FP: false-positive, FN: false-negative

© 2020 Okada Y et al. *JAMA Network Open.*

eTable 5. Diagnostic Ability of the Score Group in Validation Cohort

Group (score)	Sp	Se	LR+	LR-	TP	TN	FP	FN
High (3-4)	0.810	0.491	3.35	0.73	28	325	76	29
Middle (2)	0.399	0.895	1.49	0.26	51	160	241	6
Low (1)	0.060	0.982	1.04	0.29	56	24	377	1
Very Low (0)	-	-	-	-	57	0	401	0

Sp: Specificity, Se: Sensitivity, LR+: Positive likelihood ratio, LR-: Negative likelihood ratio, TP: true-positive, TN: true-negative, FP: false-positive, FN: false-negative

© 2020 Okada Y et al. *JAMA Network Open.*

eFigure 2. Decision Curve Analysis

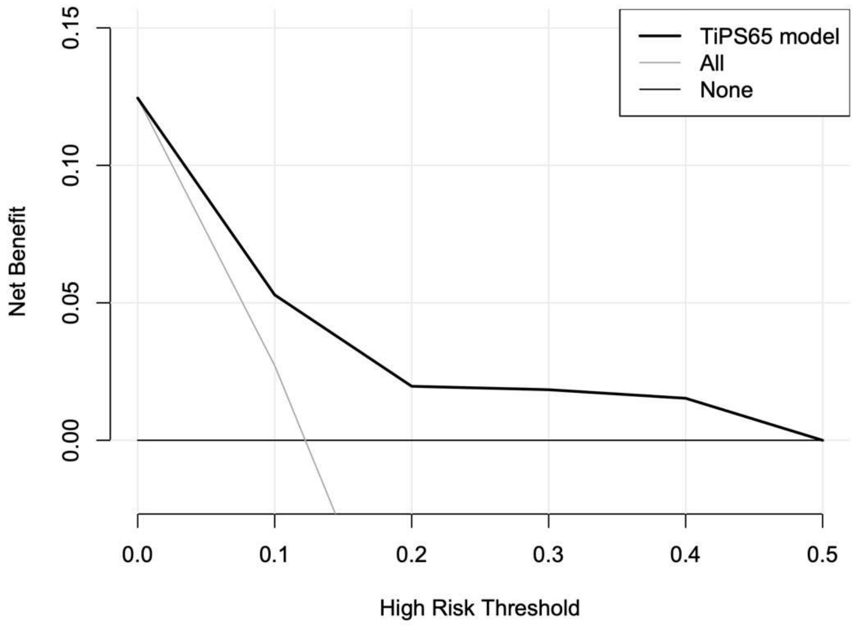

X-axis: Threshold probability, Y-axis: Net-benefit. The net-benefit of using the TiPS65 scoring system is higher than that when ECPR is performed in all patients. In this analysis, we either assumed all patients performed ECPR (All ECPR strategy) as actual situation or performed no ECPR and unfavorable outcome regardless of the score. The detail of decision curve analysis is described in S-Appendix 1.

© 2020 Okada Y et al. *JAMA Network Open.*

eReferences

1. Cummins RO, Chamberlain DA, Abramson NS, et al. Recommended guidelines for uniform reporting of data from out-of-hospital cardiac arrest: the Utstein Style. A statement for health professionals from a task force of the American Heart Association, the European Resuscitation Council, the Heart and Stroke Foundation of Canada, and the Australian Resuscitation Council. *Circulation.* 1991;84(2):960-975.
2. Fitzgerald M, Saville BR, Lewis RJ. Decision curve analysis. *Jama.* 2015;313(4):409-410.
3. Vickers AJ, Van Calster B, Steyerberg EW. Net benefit approaches to the evaluation of prediction models, molecular markers, and diagnostic tests. *Bmj.* 2016;352:i6.
4. Vickers AJ, van Calster B, Steyerberg EW. A simple, step-by-step guide to interpreting decision curve analysis. *Diagnostic and Prognostic Research.* 2019;3(1):18.
5. Steyerberg EW. *Clinical prediction models : a practical approach to development, validation, and updating.* Vol: hardcover. New York ; London: Springer; 2009.

© 2020 Okada Y et al. *JAMA Network Open.*
